# Evaluation of Anti-Tumorigenic Effects of Diosmetin against Human Colon Cancer Xenografts in Athymic Nude Mice

**DOI:** 10.3390/molecules24142522

**Published:** 2019-07-10

**Authors:** Sanaz Koosha, Zahurin Mohamed, Ajantha Sinniah, Mohammed A. Alshawsh

**Affiliations:** Department of Pharmacology, Faculty of Medicine, University of Malaya, Kuala Lumpur 50603, Malaysia

**Keywords:** colon cancer, diosmetin, anti-tumorigenesis, apoptosis, nude mice

## Abstract

Colon cancer is the third most common type of cancer in the world. Diosmetin (Dis), a natural *O*-methylated flavone, has been reported to have anti-cancer effects against different types of cancer. Although the mechanisms of action of Dis against several cancer cell lines are well reported, in vivo anti-tumorigenesis properties of this compound are still obscure. Therefore, this study aimed to investigate the anti-tumorigenesis properties of Dis against HCT-116 colon cancer xenografts in nude mice. HCT-116 colon cancer cells were injected in NCr nu/nu nude mice and treatment with Dis was initiated after the tumor volumes reached 100 mm^3^ and continued for four weeks. On the sacrificing date nude mice treated with 100 mg/kg of Dis showed significant lower tumor volume (264 ± 238.3 mm^3^) as compared to the untreated group (1428.8 ± 459.6 mm^3^). Anti-apoptotic Bcl-2 protein was significantly downregulated, while apoptotic protein (Bax) was significantly overexpressed in nude mice treated with 100 mg/kg Dis as compared to untreated mice. In conclusion, our in vivo results indicate that Dis significantly reduces tumor growth rate of HCT-116 colon cancer cells in nude mice at a dose of 100 mg/kg, and has no toxic effects in ICR mice up to 2000 mg/kg.

## 1. Introduction

Colon cancer (CRC) is the third leading cause of cancer mortality in the world with increasing trends in incidence and mortality rate [1]. Current chemotherapy drugs for CRC such as oxaliplatin and 5-fluorouracil (5-Fu) come with a variety of undesirable side effects such as diarrhea, bone marrow suppression, peripheral neuropathy and cardiotoxicity [2]. In recent decades, natural products have attracted attention as potent candidates to combat cancer due to their anti-cancer efficacy and low side effects [3].

Diosmetin (Dis) is an *O*-methylated flavone that can be extracted from citrus and other medicinal plants. Dis inhibits proliferation of several cancer cell types such as hepatocarcinoma, leukemia, breast, lung and prostate [4,5,6,7,8]. Dis inhibits cell cycle progression in mitosis phase due to suppression of polo-like kinase 1 (PLK1) factor [4]. Several studies have confirmed that proliferation of A549, MDA-MB 468, LNCaP and PC3 cancer cells are inhibited by Dis at G0/G1 phase of the cell cycle [5,6,7,8]. In addition to inhibition of cell proliferation and metastasis, Dis inhibits matrix metalloproteinases (MMP 2 and MMP 9) in hepatocellular carcinoma (HCC) cells [9].

Other than inhibition of metastasis and cell proliferation, Dis induces apoptosis in prostate cancer cells through over expression of Bax, p27kip1 and Foxo3 and down regulation of Bcl-2 and c-Myc [6]. Induction of apoptosis in leukemia and hepatocarcinoma cells occur through activation of extrinsic apoptosis pathway and inhibition of NF-κB and activation of p53, respectively [8,10,11]. Dis showed cytotoxic effects against various colon cancer cell lines such as Caco-2, HT-29 and Colo205 [12,13,14]. The study was carried out in our lab to assess the mechanistic signaling pathways underlying the anti-proliferative and apoptosis properties of Dis against HCT-116 colon cancer cells (in vitro). This demonstrated that Dis mediates the disruption of mitotic associated genes and cyclin A/B and induces apoptosis via inhibition of NF-κB translocation and upregulation of apoptotic factors including Fas and Bax of Dis on different cancer cell lines, the anti-tumorigenesis properties of Dis in an animal model needs to be explored to confirm in vitro findings. In the present study, the in vivo safety level of Dis was investigated through acute toxicity study. In addition, the anti-tumorigenic activity of Dis was evaluated against human colon cancer cells (HCT-116) xenografts in nude mice.

## 2. Results

### 2.1. Acute Toxicity of Diosmetin

**Figure 1:**
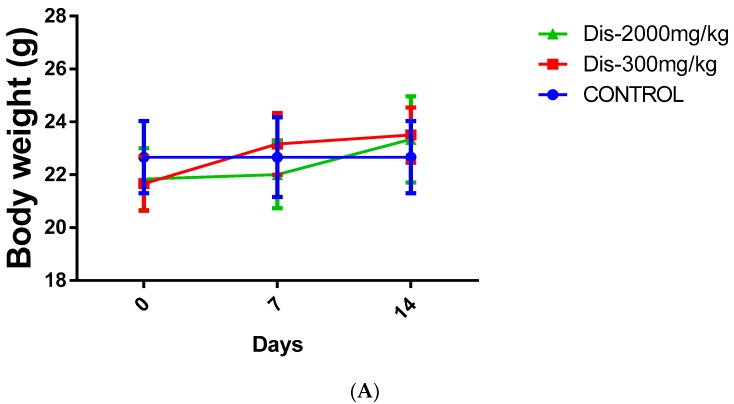

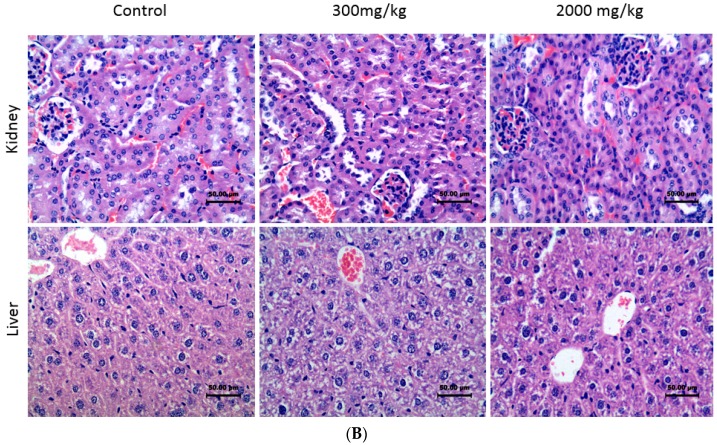
Toxicity effect of Dis in female ICR mice. (**A**) Body weight changes of mice between treated mice and untreated control during 14 days. Data were expressed as mean ± standard deviation. (**B**) Toxicity effect of Dis on histological sections of liver and kidney of mice. Mice were treated with single dose of vehicle (control) or Dis (300 and 2000 mg/kg). Kidney and liver of ICR mice were harvested after 14 days and stained with Hematoxylin and Eosin (H&E) staining. No sign of kidney or liver tissues damage or abnormalities were observed in treated and control groups. (Magnification ×40).

The acute toxicity test of Dis on female ICR mice did not demonstrate any signs of toxicity or mortality in the treated animals during the 14 days of observation after the oral administration of a single dose of 300 mg/kg and 2000 mg/kg of Dis. Moreover, no abnormal behavior such as changes in eyes, fur, skin, respiration, sedation, and convulsion were observed within 14 days. There were no significant changes in body weight between treated and untreated mice (Figure 1A). Furthermore, no abnormality was observed in the histopathology of the liver or the kidney of any of the mice (Figure 1B). No signs of inflammation were observed in the liver of treated and untreated mice as demonstrated by absence of lymphocytes accumulation around the hepatic vein. Cell walls were intact in both kidney and liver tissues and no signs of necrosis were observed. Kidney glomeruli were normal and no sign of kidney damage was observed in treated and control groups (Figure 1B).

### 2.2. Dis Reduces Tumor Size in HCT-116 Colon Cancer Xenograft Nude Mice

**Figure 2:**
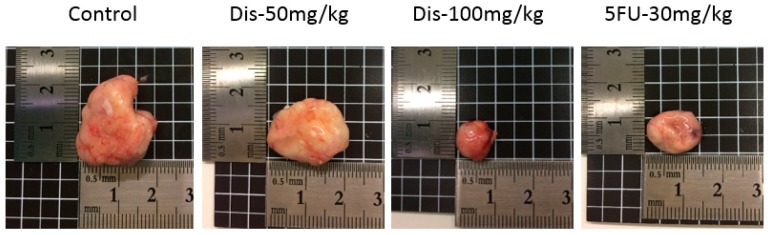
Tumor size of treated and untreated groups. Dis reduces the tumor size dose-dependently. 5-fluorouracil was used as a positive control.

**Figure 3:**
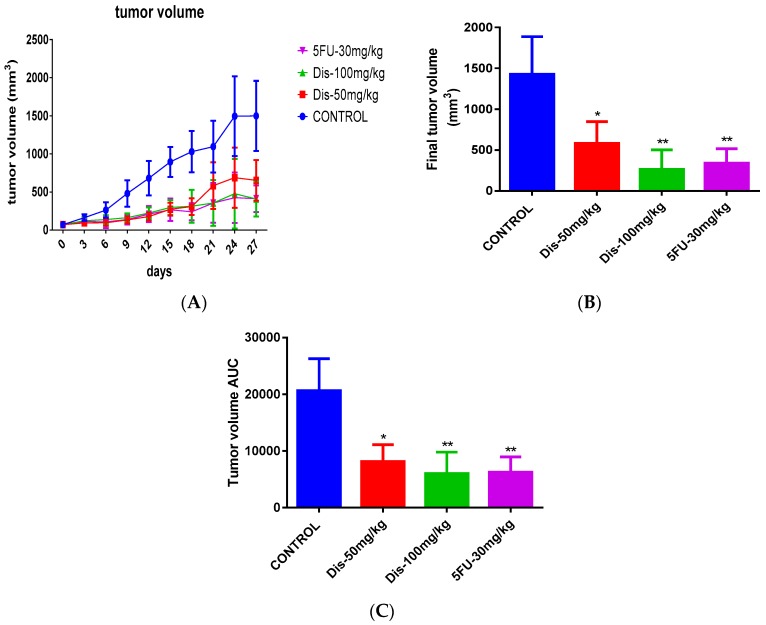
Tumor volume growth and final tumor volume of treated and untreated mice. (**A**) Tumor volume growth in control and treated groups during 27 days of treatment. (**B**) Final tumor volume of treated and untreated groups. (**C**) Area under the curve (AUC) measurement of the overall changes in tumor volume of treated mice compared to untreated group. Data were expressed as mean *±* standard deviation. * *p* < 0.05, ** *p* < 0.01 indicate significant differences compared to control.

To investigate the anti-tumorigenic properties of Dis, HCT-116 cancer cells were injected into the right flank of Ncr nu/nu mice subcutaneously. After the tumor volume reached 100 mm^3^, mice were orally administered with vehicle or Dis at dose of 50 and 100 mg/kg of body weight for four weeks. The positive control group received 30 mg/kg of 5-Fu intraperitoneally as a standard drug. Results of the tumor tissue indicate that Dis suppress the development of tumor in nude mice. As shown in Figure 2 the tumor size of Dis and 5-Fu treated mice were smaller as compared to the untreated group. Final tumor volume on the sacrificing date of 5-Fu, 50 mg/kg and 100 mg/kg of Dis were 340 ± 175.5 mm^3^, 581.7 ± 266 mm^3^ and 264 ± 238.3 mm^3^, respectively, which were significantly smaller as compared to the untreated group (1428.8 ± 459.6 mm^3^) (Figure 3A,B). Area under the curve (AUC) of each group was measured and compared to the untreated group in order to monitor the tumor growth rate during the 27 days of treatment. As shown in Figure 3C, treatment with 50 mg/kg and 100 mg/kg of Dis significantly reduced tumor volume growth rate from 20647 ± 5653 in control group to 8131 ± 2988 (*p* < 0.05) and 6009 ± 3788 (*p* < 0.01), respectively. AUC of 5-Fu group showed that tumor growth rate of mice treated with the standard drug (6270 ± 2698) was slightly higher as compared to the high dose of Dis (100 mg/kg).

Based on the tumor ratio percentage formula, 100 mg/kg of Dis has the lowest tumor growth (22.3%), followed by 5-Fu group (27.4%) and 50 mg/kg of Dis (43.5%). These data suggest that Dis inhibits tumor growth rate dose-dependently.

**Figure 4:**
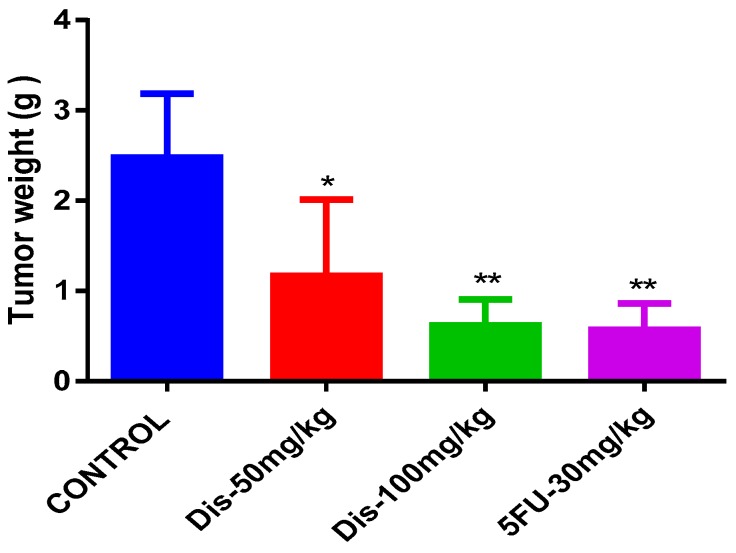
Tumor weight of treated and untreated mice. Tumor weight in grams after 27 days of treatment with Dis compared to control. Data were expressed as mean ± standard deviation. * *p* < 0.05, ** *p* < 0.01 indicate significant differences compared to control.

On the other hand, tumor weight of the mice was measured and statistically compared between the treated and untreated groups. As presented in Figure 4, after 27 days of treatment, tumor weights were significantly lower in all treated groups compared to untreated control. Although 50 mg/kg of Dis reduced the tumor weight (1.17 ± 0.84 g) significantly (*p* < 0.05), more reduction in tumor weight was observed in mice treated with the high dose of Dis (0.62 ± 0.29 g) (*p* < 0.01) and 5-Fu group (0.57 ± 0.28 g) compared to the untreated (2.478 ± 0.7 g) mice.

### 2.3. Apoptotic Effects of Dis in HCT-116 Xenograft Nude Mice

**Figure 5:**
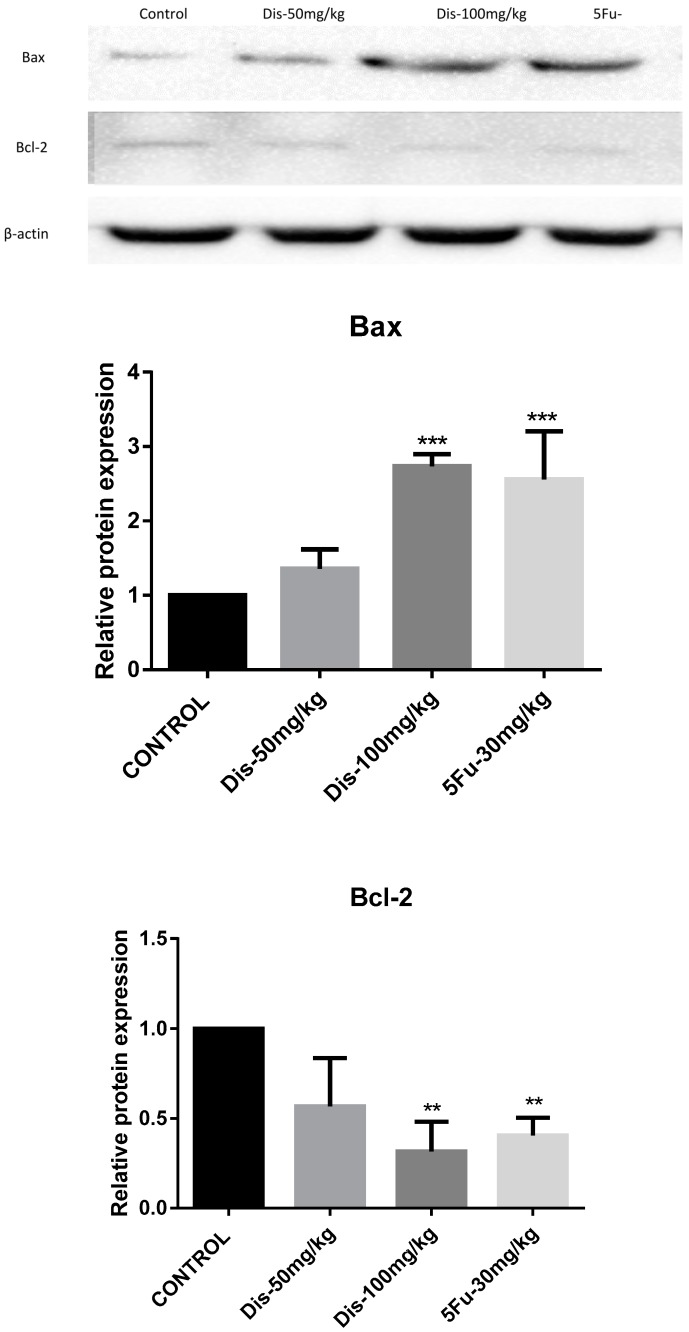
Dis altered apoptosis markers. Western blot analysis was carried out to evaluate the protein expression levels of Bax and Bcl-2. Results indicate that 100 mg/kg of Dis induces apoptosis through activation of Bax and inhibition of Bcl-2. β-Actin served as a loading control. Data were expressed as mean *±* standard deviation. ** *p* < 0.01, *** *p* < 0.001, indicate significant differences compared to control.

Xenograft tumor tissues were used to investigate the effect of Dis on apoptosis markers, Bax and Bcl-2 [15]. Protein expression fold change of Bax in 50 mg/kg and 100 mg/kg of Dis were 1.4 ± 0.26 and 2.7 ± 0.16, respectively. While protein expression fold change of Bcl-2 were 0.56 ± 0.26 and 0.31 ± 0.16 for the same doses. The data indicated that protein expressions of Bax were increased, whereas Bcl-2 protein expressions were decreased dose-dependently in Dis treated mice as compared to the untreated group. Although up-regulation of Bax and down-regulation of Bcl-2 were observed in low dose and high dose of Dis treated mice, significant differences were only observed in high dose of Dis treated mice (Figure 5). Moreover, up-regulation of Bax (2.5 ± 0.64) in 5-Fu treated mice was slightly lower than 100 mg/kg of Dis treated mice and down regulation of Bcl-2 (0.4 ± 0.09) in 5-Fu group was slightly higher compared to the high dose of Dis.

## 3. Discussion

In this study, we investigated the anti-tumorigenesis effects of Dis, and findings showed that Dis inhibits tumor growth rate through induction of apoptosis in nude mice via overexpression of Bax and inhibition of Bcl-2. Daily administration of 100 mg/kg of Dis for 27 days reduced tumor ratio percentage by 22.3% in xenograft mice. Data of animal study showed that Dis reduces the tumor growth rate and tumor weight significantly in dose-dependent manner, however protein expression changes of Bax and Bcl-2 significantly observed only in high dose of Dis treated mice. In addition, 100 mg/kg of Dis inhibits tumor growth rate slightly better compared to the 5-Fu, however these differences were not significant. Acute toxicity results indicated that Dis is safe up to 2000 mg/kg no signs of toxicity were observed in the kidney or liver of ICR mice.

Based on tumor ratio percentage formula, the percentage of reduction in tumor growth rate among treated groups has the following order; 100 mg/kg Dis > 30 mg/kg 5-Fu > 50 mg/kg Dis. Moreover, on the sacrificing date the mean tumor weight in mice treated with 50 mg/kg, 100 mg/kg and 5-Fu were 1.17 ± 0.84, 0.62 ± 0.29 and 0.57 ± 0.28 g, respectively. Although tumor growth rate and tumor size was lower in high dose Dis treated mice as compared to other treated groups, tumor weight of the 5-Fu group was slightly lower compared to other groups. Even with these differences between the treatment groups, significant changes in tumor volume and tumor weight were only observed when compared to the control group.

Internal or external stimuli can induce cell programmed death or apoptosis. Targeting apoptosis signaling pathway in cancer cells is considered one of the strategies in drug discovery to explore the molecular mechanisms of potent anti-cancer agents. These agents can induce apoptosis through alteration of apoptotic proteins such as Bax, which is located in the outer membrane of mitochondria. Bax protein increases the permeability of mitochondria membrane which leads to release of other apoptotic factors and cytochrome C, whereas inhibition of anti-apoptotic proteins such as Bcl-2 blocks their action and further enhances the cell proliferations. Release of apoptotic factors such as cytochrome C leads to caspase cascades activation and initiation of apoptosis [16]. Based on our in vitro study, Dis was observed to inhibit HCT-116 cell proliferations and induce apoptosis through intrinsic and extrinsic pathways. Dis was also observed to upregulate Bax at both gene and protein levels. As a result, intrinsic apoptotic pathway was initiated due to the release of cytochrome C into the cytosol fallowed by cleavage of caspase-9. Extrinsic apoptosis pathway was also activated in Dis treated cells with cleavage of caspase-8 via dead domain of TNF-α/Fas. Furthermore, Dis caused acceleration of apoptosis through inhibition of NF-κB translocation into the nucleus [17].

Flavones are a subclass of flavonoids and are mainly found in chamomile, parsley, rosemary, rooibos tea, green tea, and other plants from the mint family (Lamiaceae) [18]. The most common flavones include apigenin, luteolin and diosmetin. Based on the literature, flavones may exert their anticancer effects through several possible mechanisms, such as scavenging of carcinogenic agents, modulation of cancer cell signaling, antioxidant enzymatic activities, and induction of apoptosis as well as cell cycle arrest [19]. Therefore, investigation into the anti-cancer properties of flavones as a member of flavonoids family has been attracting the attention of researchers. Besides their therapeutic potential, since flavones are present in our diet, a greater understanding of their anticancer properties might also modify our dietary habits as a step to be taken to prevent cancer.

Anti-cancer properties of Dis have been reported in several types of cancer cells. Dis induces apoptosis in PC-3 human prostate cancer cells through overexpression of Bax, P27kip1 and Foxo3 and downregulation of Bcl-2 and c-Myc [6]. Dis does not only inhibit prostate cancer cells via apoptosis but it also inhibits hepatocarcinoma and leukemia cells. Dis induces extrinsic apoptosis pathway in leukemia, whereas induction of apoptosis in hepatocarcinoma is through activation of p53 and inhibition of NF-κB translocation [4,5,6,7,8,9,10,11].

Previous studies showed that extracts of different plants, which contain Dis such as Anoda cristata, Fructus aurantii immaturus, Satureja khuzistanica and South American Tanacetum vulgare has in vivo anti-inflammatory and antioxidant properties [20,21,22,23]. Moreover, pretreatment of Dis attenuates acute hepatic failure in mice induced by LPS/D-GalN endotoxin through inhibition of apoptosis, inflammation and oxidative stress. In addition, Dis inhibits the phosphorylation of IκBα, and NF-κB p65 in the NF-κB signaling pathway. Pretreatment of Dis can also increase the level of radical-scavenging antioxidant enzymes such as superoxide dismutase (SOD) and catalase (CAT) in mice [24]. Yang, et al., (2017) reported that, pretreatment of Dis may protect the normal liver cells against endotoxins by inhibiting inflammation and oxidative stress factors, therefore normal cells do not enter the apoptosis and cell programmed death process.

Our in vivo results showed that the protein expression of Bax was significant increased, whereas protein expression of Bcl-2 was down-regulated in high dose Dis treated mice as compared to the control group. Therefore, we suggest that reduction in tumor size and weight of Dis treated mice could be as a result of apoptosis via activation of Bax and inhibition of Bcl-2 proteins, respectively.

## 4. Materials and Methods

### 4.1. Compound and Cell Lines

**Figure 6:**
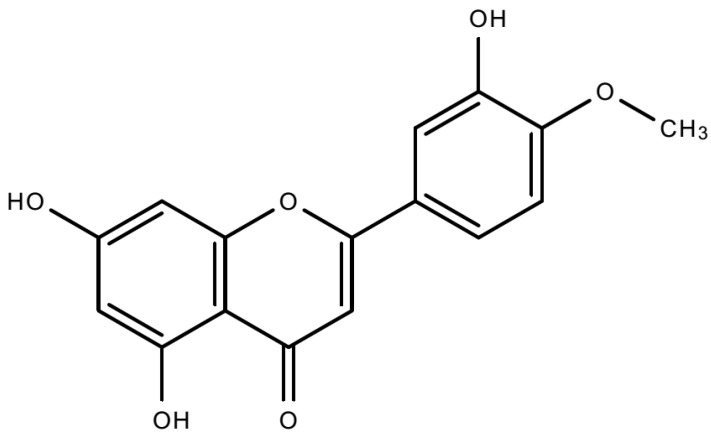
Chemical structure of Diosmetin.

Diosmetin was obtained from Abcam (Cambridge, UK) with the purity >98% and molecular weight of 300.26 g/mol (Figure 6). 5-Fluorouracil (5-Fu) was purchased from MP biomedical company (MP biomedical, Illkirch-Graffenstaden, France). Human colon cancer cells (HCT-116) was obtained from ATCC, USA.

### 4.2. Acute Toxicity

Oral acute toxicity test was performed according to OECD guideline No 423 [25,26]. All animal experimental procedures were carried out according to the approval of the institutional Animal Care and Use Committee, Faculty of Medicine (FOM-IACUC), (Ethics reference No.: 2017-181205/PHAR/R/SK). The animal experiment was conducted as a stepwise procedure with the use of three female ICR mice per dose. Absence or presence of compound-related mortality of the animals dosed at each step determined the following step of either dosing of three additional animals with the same dose or dosing of three additional animals at the next higher or the next lower dose levels. Since there is no information on the toxicity of Dis, 300 mg/kg body weight was used as the starting dose according to OECD 423 guideline [25,26]. In total, a maximum of 18 female ICR mice (4–6 weeks old, 20–24 g) were used in this experiment and the grouping was as follows: Group 1: normal control, which received distilled water orally, Group 2: 300 mg/kg Dis was administered as a single dose orally, Group 3: next higher dose level of Dis (2000 mg/kg) was administered as a single dose orally. Animals were observed closely for 14 days. All the animals which survived to the end of the experiment were sacrificed on day 15th under anesthesia using proper doses of ketamine (100 mg/kg) and xylazine (10 mg/kg). Animal weights were measured and liver and kidney were harvested for histopathological examination to evaluate signs of toxicity.

### 4.3. Human Colorectal Cancer Xenografts in Athymic Nude Mice

A total of 24 male NCr nu/nu nude mice (4–6 weeks old, 20–22 g) were purchased from an external source with health certificate (Sterling, Singapore). Colorectal cancer HCT-116 cells (2 × 10^6^ cells) in 200 µL of media were subcutaneously injected into the right flank of mice. After the volume of the tumors reached 100 mm^3^ [27], treatments were started by utilizing oral gavage for 4 weeks. Animals were randomly assigned to the following four groups namely; Group 1: tumor control (orally treated with the vehicle 0.5% CMC), Group 2: orally treated with Dis at 50 mg/kg (low dose) daily, Group 3: orally treated with Dis at 100 mg/kg (high dose) daily and Group 4: positive control (intraperitoneally injected with 30 mg/kg 5-Fu once per week) [27]. Tumor growth were measured two times per week using a digital caliper. Area under the curve (AUC) for the mean tumor volume versus days for each group were calculated using Graph Pad Prism, version 6.07 (Graph Pad, San Diego, CA, USA) for windows [28,29,30].

In order to understand the anti-tumor activity of Dis and to have a better comparison between treated and untreated mice, tumor ratio percentage was calculated according to the following formula [31]:

Tumor ratio precentage=T/C×100,

where, T is the mean tumor volume of treated mice on the sacrificing date, and C is the mean tumor volume of control group.

On the sacrificing date, ketamine (100 mg/kg) and xylazine (10 mg/kg) were injected as a single dose intraperitoneally (IP). The tumors were harvested, washed and weighted. Tumor samples were kept at −80 °C for western blot assay.

### 4.4. Western Blot

Lysis buffer (50 mM Tris-HCL pH 8.0, 120 mM NaCl, 0.5% NP-40, 1 mM PMSF) was added to the tumor specimens and homogenized using homogenizer (Cole-Parmer, USA). Lysates were then centrifuged for 30 min at 15,000× *g* and 4 °C. Supernatant was collected and the protein concentration was quantified via Pierce BCA protein assay kit (Thermo Scientific™, Pittsburgh, PA, USA) [32]. An amount of 80 µg of protein were separated by 10% SDS PAGE, then transferred to a polyvinylidene difluoride (PVDF) membrane (Bio-Rad, USA), then were blocked with 3% skim milk in TBS-Tween buffer 7 (0.12 M Tris-base, 1.5 M NaCl, 0.1% Tween 20) for 1 h at room temperature, and were incubated with the primary antibody (β-actin (1:10,000), Bcl-2 (1:1000) and Bax (1:1000)) for 2 h. Then horseradish peroxidase conjugated secondary antibody was added and incubated for 2 h at room temperature. The bound antibody was detected with peroxidase-conjugated secondary antibody followed by chemiluminescence detection (ECL System) (Bio-Rad, Hercules, CA, USA) and exposed by UVP (Bio-Rad, Hercules, CA, USA).

### 4.5. Statistical Analysis

All the data were expressed as mean ± standard deviation (SD) and statistical analyses was performed using one-way analysis of variance (ANOVA) with Tukey’s multiple comparisons. Data were analyzed with Graph Pad Prism, version 6.07 for windows. The values of *p* < 0.05 were considered significant.

## 5. Conclusions

In conclusion, Dis is a potent anti-tumor compound, which acts by targeting apoptosis and inhibiting colon cancer tumor growth in nude mice. Our results indicated that Dis is able to reduce the tumor weight and tumor growth rate in nude mice and is safe up to 2000 mg/kg. Moreover, overexpression of Bax and suppression of Bcl-2 illustrates that the mechanism of tumor suppression of Dis might be through induction of apoptosis. These results need further pharmacokinetic and pharmacodynamics studies as well as further toxicity evaluation in order to assess whether Diosmetin could be introduced as a therapeutic agent against human colon cancer with a relatively safe profile.
